# Trends in radiation oncology between 2015 and 2022: report from a German university radio-oncology department

**DOI:** 10.1007/s00066-026-02541-0

**Published:** 2026-09-11

**Authors:** M. Diefenhardt, D. Imhoff, N. Tselis, C. Rödel, D. Martin

**Affiliations:** 1 Department of Radiotherapy and Oncology, Goethe University Frankfurt, University Hospital, Theodor-Stern-Kai 760590, Frankfurt, Germany; 2 Frankfurt Cancer Institute (FCI), Goethe University Frankfurt, Frankfurt, Germany; 3 German Cancer Research Center (DKFZ), Heidelberg, Germany, and German Cancer Consortium (DKTK), Partner Site Frankfurt, Frankfurt, Germany

**Keywords:** Radiotherapy, COVID pandemic, Demographic change, Empirical health research, Brachytherapy

## Abstract

**Purpose:**

How have the demographics of radiotherapy patients changed between 2015 and 2022? What was the impact of the pandemic, of modified multidisciplinary therapy concepts, and of structural changes on our patient cohort?

**Methods:**

We analyzed the data of all patients who underwent computed tomography-guided radiotherapy or ultrasound-planned high dose rate prostate brachytherapy at our department between 2015 and 2022. We examined tumor diagnoses in terms of frequency, age of the patients and the distance between patientsʼ place of residence and our department.

**Results:**

A total of 8842 patients underwent radiotherapy at our clinic between 2015 and 2022. The median age at treatment increased from 62 years in 2015 to 64 years in 2022. Within the entire cohort, 18% underwent metastasis-directed radiotherapy, 13% had radiotherapy for head and neck tumors, and 12% for breast or prostate cancer. Skin cancer treatments increased in frequency by 79% during study period, while prostate treatments rose by 10%. Overall, 47% of all patients lived within a 10-km radius of our clinic. Patients who received brachytherapy for cervical or endometrial cancer travelled significantly further. During the national epidemic emergency (03/2020 to 11/2021), there was no significant change in the long-term average number of patients or the proportion of cancer types.

**Conclusion:**

There has been a significant increase in radiotherapy treatments for skin cancer, alongside an aging patient population. Although we primarily act as a local radiotherapy partner, the increased travel distances for brachytherapy patients underscore the particular responsibility of a university radiotherapy department as a primary center and key provider of comprehensive cancer care.

## Introduction

Even in the era of immunotherapy and targeted oncology treatments, radiotherapy remains a key part of modern treatment protocols, with approximately half of all oncology patients undergoing radiotherapy at some point [1]. Although the role of radiotherapy is constantly being re-evaluated in light of new clinical trial results for specific cancer types or stages, its use as a neoadjuvant, adjuvant, curative or palliative treatment remains undisputed within various multidisciplinary oncological approaches [2]. Moreover, radiotherapy maintained its indispensable role in organ preservation strategies for patients with head and neck or rectal cancer [3, 4]. Furthermore, the results of late clinical trials reveal a new potential benefit of radiotherapy for patients with oligometastatic disease [5]. Obviously, these changes need to be incorporated into clinical practice, which can be challenging. Not only has substantial bureaucracy in Europe led to a drop in the number of oncology trials, but a shortage of medical personnel and financial constraints across the continent also limits the capacity of radiation departments and their ability and flexibility to adapt to new innovations [6]. In Germany, despite the need to adapt to new treatment techniques and indications, radiotherapy departments, like all clinical departments, have to deal with the consequences of a federal government initiative to restructure the German health system. This initiative aims for a more centralized and specialized clinical structure that may change treatment capacity and referral politics in coming years [7].

In light of the evolving landscape, we conducted a retrospective analysis of our patient cohort treated between 2015 and 2022. This is part of our broader attempt to provide an outcome-focused quality report in the coming years.

Our aim was to analyze trends in demographics and cancer types within our patient cohort. Furthermore, given the disruptive impact of the COVID pandemic on health systems worldwide in 2020 and the following years, we examined the extent to which it affected our department regarding patient capacity and referral structure [8]. Finally, we examined our department’s catchment area in order to analyze the distances travelled by patients for treatment and to identify cancer types that are specifically referred to our department due to our specialized expertise, which may not be available from other local or regional partners.

## Methods

Subsequent to the approval of the ethics (2023–1441) and data protection boards at Goethe University Frankfurt, raw data were exported from the primary digital clinical database that enumerates all planning computed tomography (CT) scans registered between January 2015 and December 2022. The hospital’s central data integration center then provided an extraction of all clinical cases associated with the Department of Radiotherapy and Oncology from the same time period. This information was paired with the list of planning CT scans, after excluding patients who: already had a planning CT scan in the same calendar year; had a planning CT scan but were treated at the cooperative, but not associated CyberKnife Center Frankfurt, as the ethics and data protection board approval did not cover these patients; had a planning CT scan but did not start treatment, as the reasons for this were not analyzed further. For patients who underwent both an initial planning CT scan and a restaging CT scan, the date of the initial planning CT scan was used as the reference point for the start of treatment. Only the first treatment session for each patient was counted in the primary study analysis, which did not incorporate data on multiple treatment sessions or different treatment indications per year. Data on the subcohort of patients with multiple treatment sessions in 1 year are reported separately. Patients with prostate cancer treated with ultrasound-planned brachytherapy were manually added to the database. After an initial data check, we found that in a significant number of cases (> 5%), the ICD codes specified in the raw data did not match the treatment indication.

For example, a patient with prostate cancer receiving metastasis-directed radiotherapy (C79) had been assigned a prostate code (C61). We therefore conducted a case-by-case assessment of the data, extracting the treatment indication from the final medical report of our radiation department. In cases where there was a deviation from the ICD code in the raw data, we cross-checked with the tumor conference report. Inconclusive cases were additionally discussed between M.D. and D.M.

Catchment area analyses were conducted using publicly available data based on the German postal code system. The distances were identified using ZIPCodeSoft, developed by Infotec Multimedia Solutions Ltd (Mill Mall, Suite 6, Wickhams Cay 1, Road Town, Tortola British Virgin Islands). The distance calculations were based on orthodromic (spherical trigonometric) calculations. Catchment area visualization and data visualization were performed using R statistical software and Excel®. These analyses were only performed for patients with German postal codes.

## Results

A total of 16,056 CT scans for planning purposes were performed between 1 January 2015 and 31 December 2022. Through the clinical record system, a total of 18,466 individual patient cases were identified and provided by the Data Integration Centre. Primary data sources were processed as described in the Methods section. We identified 9658 patients who underwent CT-planned radiotherapy during the study period. Of these patients, 8% (*n* = 816) underwent two or more treatment sessions in different calendar years during the study period, resulting in 8842 individual patients treated from 2015 to 2022.

**Fig. 1:**
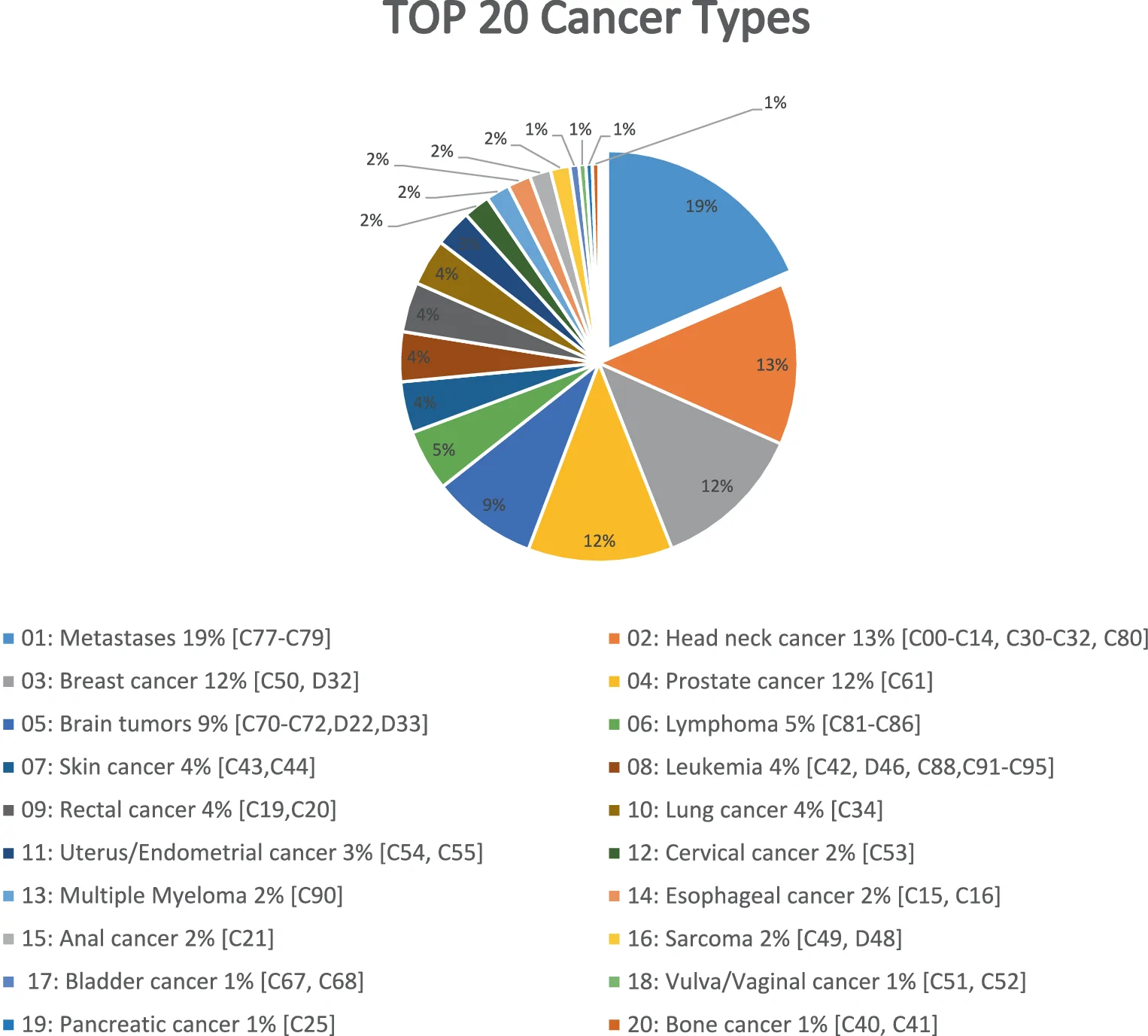
The 20 most common types of cancer treated at our department listed in order of percentage. The assigned ICD codes are listed in brackets for each cancer type.

**Fig. 2:**
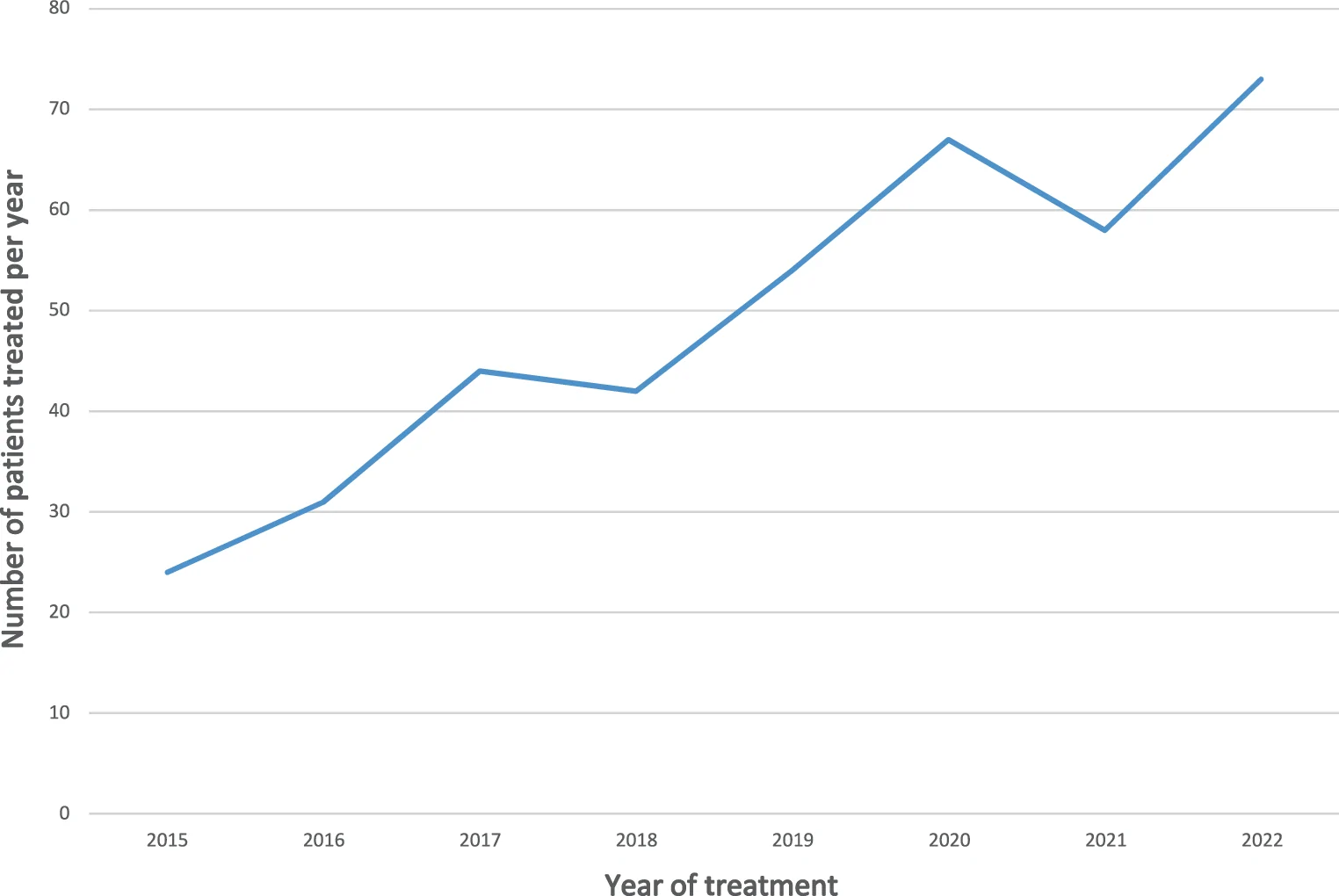
Trends in skin cancer treatments between 2015 and 2022: annual number of patients treated for skin cancer at our department.

**Fig. 3:**
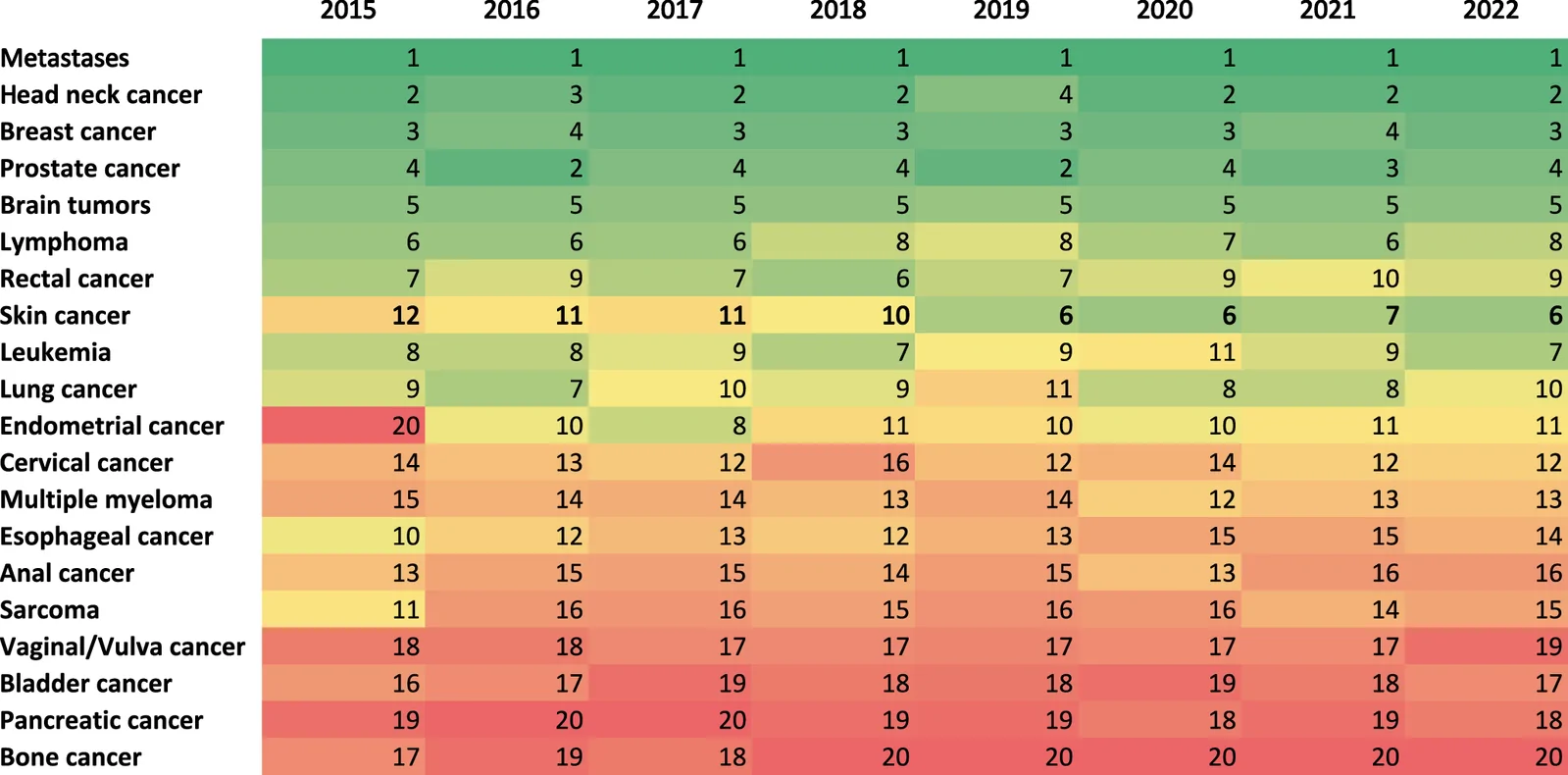
Trends in frequencies of cancer types between 2015 and 2022. The 20 most common cancer types are reported in order of their proportion during the study period. For each year during the study, the proportion of cancer types is displayed using a color scale (green indicates a higher frequency, red indicates a lower frequency). The red arrows indicate the frequency of skin and esophageal cancers in 2022.

The total number of patients treated each year remained relatively constant throughout the study period, with a median of 1226 patients per year (range: 1060 in 2015 [minimum] and 1260 in 2021 [maximum]). During this period, metastasis-directed radiation therapy accounted for 18% of all treatment sessions, followed by head and neck cancer treatments (13%), breast cancer treatments (12%), and prostate cancer treatments (11.5%). Figure 1 reports the top 20 treatment indications, accounting for 98.5% of all assessable treatment sessions. During the 8‑year study period, the relative distribution of treatment indications remained relatively steady among the five most common type of cancers (metastases, head and neck cancer, breast cancer, prostate cancer, and brain tumors). However, skin cancer cases increased, with skin cancer treatments becoming the sixth most common indication in 2022, up from 12th in 2015. Conversely, the frequency of esophageal cancer cases decreased, dropping from 10th place in 2015 to 14th place in 2022. Overall, the number of skin cancer cases increased by 79% between 2019 and 2022 compared to 2015 and 2018 (Pearson correlation: 0.957, 95% confidence interval [CI]: 0.777–0.993; Fig. 2), whereas the number of esophageal cancer cases decreased by 22% over the same periods (number of cases 2015–2018: 100 vs. number of cases 2019–2022: 78). The number of breast cancer cases remained stable, whereas the number of prostate cancer cases increased by 10%. Figure 3 reports how the 20 most common cancer types changed in rank during the study period.

We further analyzed how many patients had more than one planning CT scan during a calendar year and checked the reason for the additional planning CT scan or the associated treatment indication. Overall, 2534 (26%) patients had more than one planning CT scan in one calendar year, i.e., a total of 3808 additional planning CT scans. Adaptive radiotherapy was the reason in 66% of the cases, followed by a second course of radiotherapy in 22%, and because of repeating the initial planning CT scan in 6% of cases. Other reasons for additional planning CT scan were: planning CT for total body irradiation (3%), CyberKnife (2%), or for diagnostic purpose (1%). In total, 848 patients had additional planning CT scans because of a second course of radiation within one calendar year, as in 89% of these cases the second course of radiation was indicated because of the same primary diagnosis, while 69% of cases were due to metastasis-directed radiation. In 11% of cases the second course of radiation was indicated because of a different treatment indication than the primary indication, of which 67% were metastasis-directed radiation, in 8 cases (13%) lung cancer radiation and in 2 cases (3.3%) treatment of larynx cancer; all other indications occurred only once during the study period.

In assessments of patient age, we identified a slight increase in the median age of those receiving treatment. While the median age was 62 years in 2015, it increased to 64 years by 2022. During the study period, 2% of patients were under 18 years and 1% were over 90 years of age. Clinically meaningful differences in age distribution were observed between different cancer types: The median age was 71 years for men with prostate cancer, 78 years for patients with skin cancer, compared to 46 years for patients with leukemia and 55 years for patients with brain cancer. The most common cancer types in children (aged 1–17) were leukemia (36%) and brain tumors (21%), compared to skin cancer (40%) and head and neck cancer (23%) in the very old cohort (aged 90 and over).

**Fig. 4:**
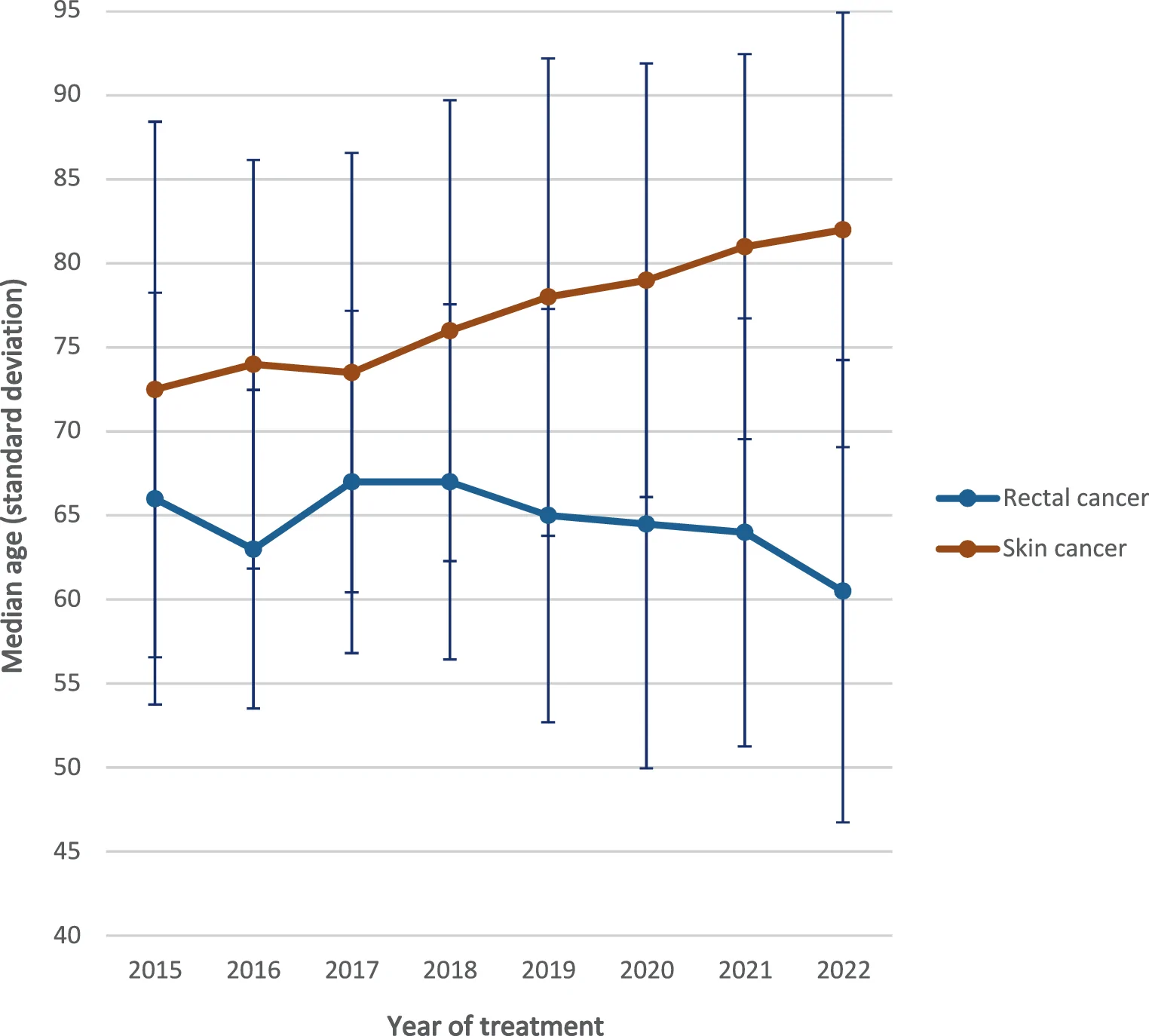
Evolving trends in age of patients with skin and rectal cancer: median age of patients with skin or rectal cancer, along with standard deviation per year (2015–2022).

While the median age of patients treated for skin cancer increased from 72.5 years in 2015 to 82 years in 2022, the median age of patients with rectal cancer fell from 66 years in 2015 to 60.5 years in 2022 (Fig. 4). Exploratory trend analyses indicate potential statistical significance in these trends: skin cancer: Pearson correlation = 0.202 (95% CI: 0.106–0.295); rectal cancer: −0.112 (95% CI −0.209–−0.012).

**Fig. 5:**
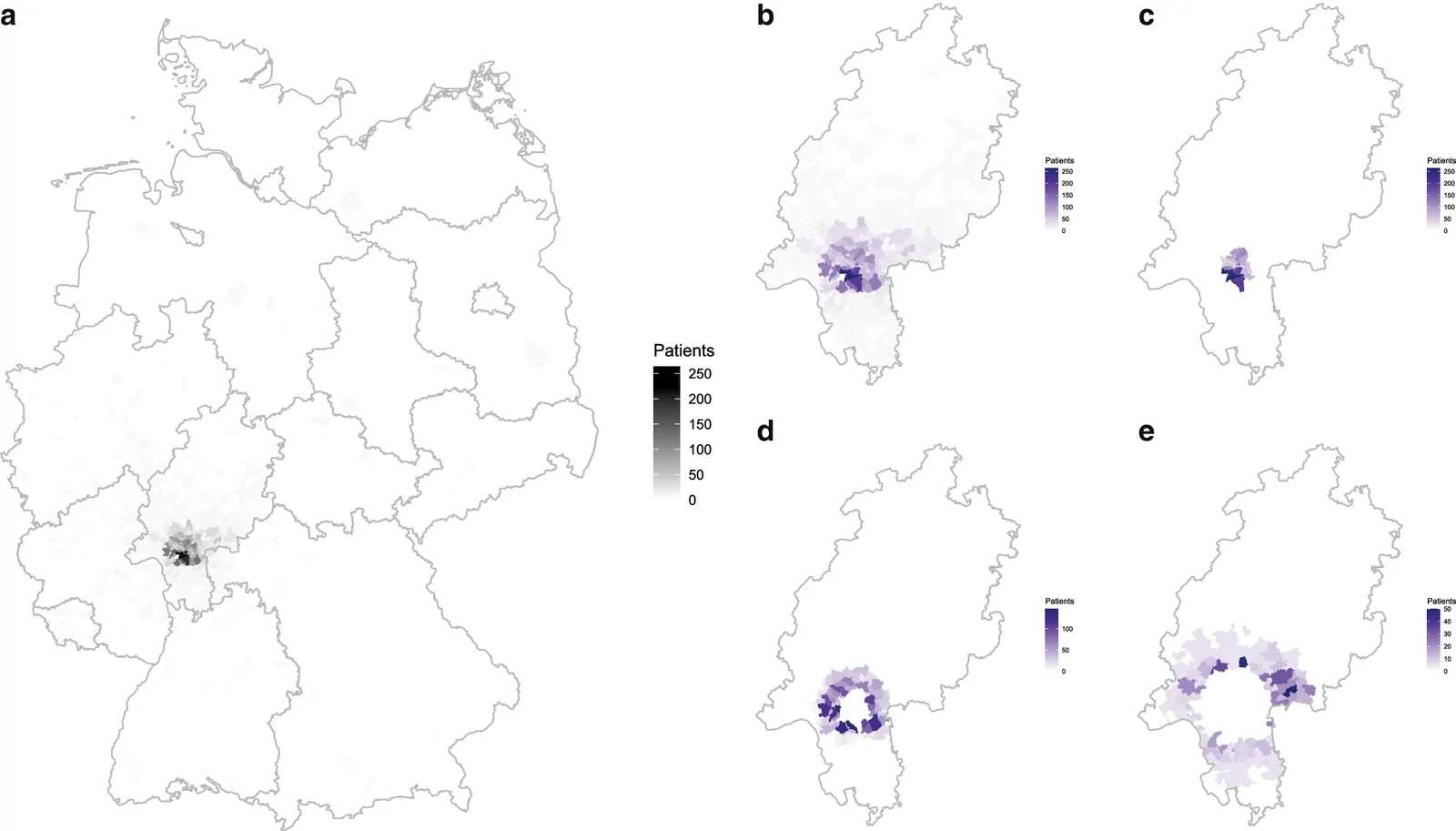
Catchment area of study cohort **a** in Germany, **b** in Hessen, **c** within 10 km, **d** within 10–25 km, and **e** within 25–50 km of our department. The corresponding absolute numbers for the color-coded frequencies are reported next to the figures (**b–e**).

The distance patients have to travel to reach radiation departments can limit their access and adherence to treatment. We therefore investigated the catchment area of our department in more detail. Figure 5 shows that our university department primarily provides local-regional access to radiation treatment. Of the 9658 individual cases between 2015 and 2022, 8769 patients with accessible postal codes were identified. At least 47% of these individuals lived within a 10-km radius of our department, 35% lived between 10 and 25 km away, 12% lived between 25 and 50 km away, and 95% lived within a 50-km radius of our department.

**Fig. 6:**
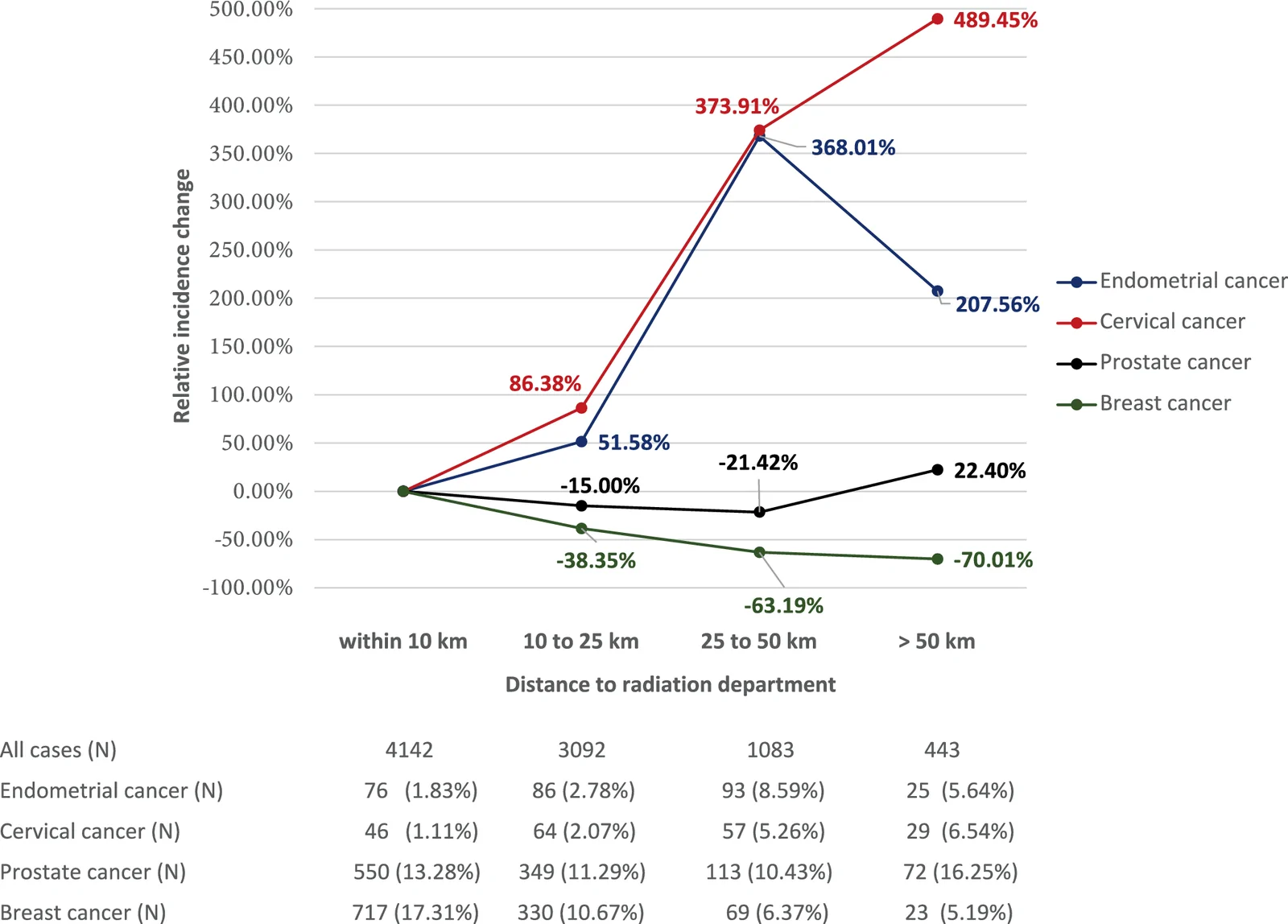
Evolving trends in the number of cases and proportion of cases of endometrial, cervical, prostate, and breast cancer depending on the distance to the department relative to the number of cases within a 0–10-km radius.

**Fig. 7:**
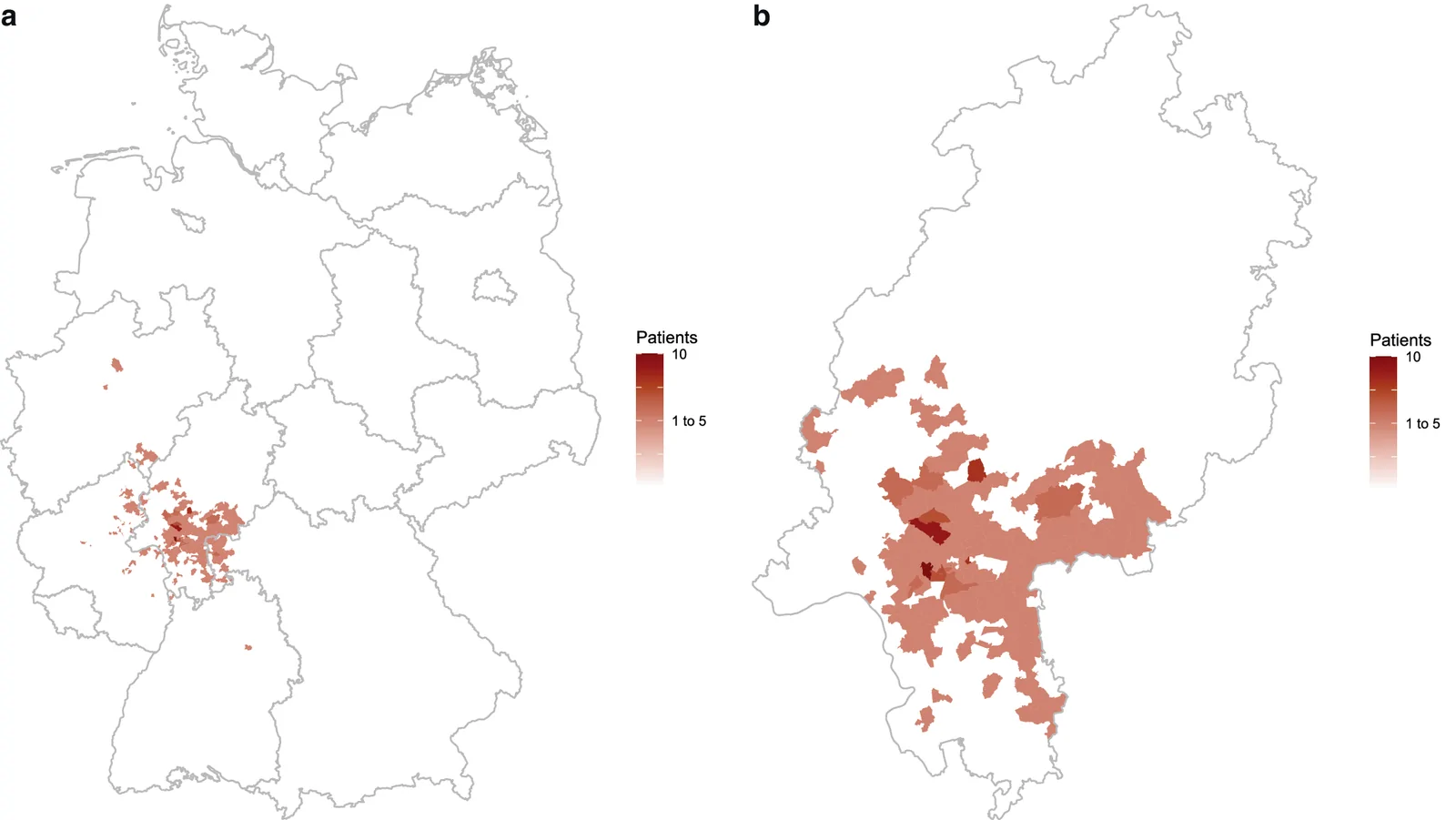
Catchment area of patients with cervical or endometrial cancer: **a** Germany **b** Hessen. The figures illustrate the large catchment area of patients with cervical and endometrial cancer, with the absolute number of patients in each postal code area reported next to the figures. In accordance with the approved data protection concept, a minimum of 5 cases are shown on the color scale in each postcode area with at least 1 case. The figure therefore only distinguishes by color those postcode areas with more than 5 cases.

The proportion of treatment entities changed significantly within the investigated distance area of our department. Whereas patients with breast cancer represented 17% of all patients within a 10-km radius, this proportion decreased to 11% within the 10–25-km area and to 6% within the 25–50-km area. Conversely, the proportion of patients with cervical and endometrial cancer increased from a combined total of 3% within a 10-km radius to 5% within a 10–25-km radius, and to 14% within a 25–50-km radius. For patients registered at a distance of more than 50 km, prostate cancer accounted for 16% of all patients, the highest proportion, followed by patients with metastases (14%) and with leukemia (11%). Figure 6 shows the relative change in the proportion of breast, prostate, cervical, and endometrial cancer in relation to patients registered within a 10-km radius, while Fig. 7 show the geographic distribution of patients with cervical and endometrial cancer treated at our department.

Between 25 March 2020 and 25 November 2021, a national epidemic emergency (Epidemische Lage von nationaler Tragweite) was declared in Germany. We analyzed whether the number of patients treated changed during this period, and how this affected the distribution of cancer types. Due to significant overdispersion in the count data (variance = 229.26, mean = 100.61, Poisson_Mod$deviance/Poisson_Mod$df.residual = 1.65, and Pearson chi-square statistic < 0.001), a negative binomial regression model was used to evaluate the effect of the pandemic on the number of patients treated per month, adjusting for seasonal trends. The regression model indicated no statistically significant effect of the pandemic on the monthly number of patients (estimate = 0.02, *p* = 0.47), as the analysis confirmed seasonal trends. Compared to January, the number of patients treated were, for example, significantly lower in February (−0.16, *p* = 0.004) or December (−0.39, *p* < 0.001). During the pandemic, 2036 patients were treated, equating to 3.3 new cases per day, or 4.8 patients per working day, compared to 3.3 new cases per day (4.8 per working day) before the pandemic and 3.2 (4.7) after.

Besides a slight drop in the number of patients treated for brain tumors (0.44 per working day before the pandemic and 0.33 during the pandemic) and an increase in the number of patients treated for skin cancer (0.16 before the pandemic and 0.28 after the pandemic), no significant changes in the proportions of cancer types were found. Compared to the periods before and after the epidemic emergency, the mean number of new patients treated per month remained constant at a median of 101 before and during the pandemic and decreased slightly to 97 afterward. However, in September/October 2020/2021, there was a drop of more than 10% in new patients compared to pre- and post-pandemic periods, although this was compensated for by a higher number of patients in December 2020 and November 2021. The catchment area was not significantly affected by the pandemic, as 47% of patients treated during the pandemic lived within a 10-km radius, 35% lived between 10 and 25 km away, 14% lived between 25 and 50 km away, and only 4.6% lived more than 50 km away.

## Discussion

Technological progress in radiation oncology has shaped the last decade. Furthermore, new clinical trials have challenged the role of radiation oncology in certain cancer types, while new applications have emerged, particularly with regard to oligometastatic situations and the potential for organ preservation through radiation therapy. Additionally, the global disruption caused by the pandemic had a significant impact on the workflow of radiation departments, leading to innovative clinical approaches such as broader implementation of hypofractionated schemas and shortened treatment protocols [9–11]. This study analyzed our patient cohort over an 8‑year period to identify changes in cancer types and/or demographics that will need to be adapted to in order to continue providing patient-centered, needs-oriented treatment approaches in the coming years.

In March 2020, the German federal government declared a national epidemic emergency, which lasted until November 2021. Even after this period, clinical daily practice was characterized by workflows that adhered to virology recommendations. Unexpectedly, neither the number of patients treated nor the proportion of individual cancer types changed significantly during the pandemic. These data suggest that, in our oncology department at least, the pandemic was not as disruptive as has been reported elsewhere, since oncological treatment remained feasible. As we could not identify a change in the catchment area during the pandemic period compared to the periods before and after, we cannot hypothesize that the oncology referral network was permanently disrupted.

Overall, we found that the number of patients treated at our department per year remained largely consistent throughout the study period. The number of linear accelerators (*n* = 3) and high dose rate (HDR) brachytherapy units (*n* = 1) did not change, and there were no prolonged staff shortages that could have affected our department’s capacity. On average, six to eight board-certified radiation oncologists and six to nine residents were employed. We identified 2534 patients (22%) who had at least one additional planning CT scan within 1 year. Of these treatments, 89% were indicated based on the primary diagnosis, and 69% underwent metastasis-directed radiation. Furthermore, metastasis-directed radiation was the differing treatment indication in 67% of additional treatments. As our main analysis only counted the first treatment session, we may have underestimated the total number and percentage of metastasis-directed radiation sessions in our study.

The relatively constant total number of patients treated at our department over the 8‑year study period indicates that the increased use of hypofractionated treatment protocols, e.g., for breast cancer, in recent years has not led to an increase in treatment capacity in our department. The broader adoption of time-consuming radiation techniques, such as breath-controlled radiotherapy in stereotactic body radiotherapy (SBRT) or for breast cancer, may be a possible explanation for the lack of increase in patient numbers during the study period. Furthermore, as a university hospital with a large pediatric oncology and stem cell transplant department, we provide complex and time-consuming techniques such as total body irradiation, and obviously performing radiotherapy on anesthetized children not only requires anesthetic coordination, but is also associated with prolonged time at the linear accelerator [12]. Whether the capacity of radiation departments could be increased by more hypofractionated regimens in, e.g., prostate cancer, with faster online adaptive treatment planning is not yet evident [13].

Our results emphasize certain broader epidemiological trends in oncology, as overall in Germany the age-standardized incidence and mortality rates for cancer decreased for women and men during the study period but the absolute number of new cancer cases and deaths increased [14]. As the median age of patients in our study cohort increased overall, we observed a worrying opposite trend in rectal cancer, which is consistent with broader epidemiological reports indicating increasing incidences in younger patients [15]. The results from the ACO/ARO/AIO-18.1 trial will hopefully indicate whether younger patients benefit from organ preservation approaches in the same way as older patients. However, the overall trend of an aging population of patients should prompt strategic questions about access to radiation therapy in Germany and Europe. Local radiotherapy departments reduce the burden of travel time and associated costs for patients, which is an important consideration as Germany’s deficit-ridden health system faces structural reforms. Conversely, a more concentrated supply of treatment could mean longer travel distances for some patients, which could be more burdensome, especially for older patients and those receiving palliative care [16, 17]. On the other hand, more centralized oncology treatment units with more local staff and capacity could enhance the quality of treatment delivery by facilitating a more specialized workforce and offering staff more flexible working hours, which could be a strategic benefit when recruiting new staff.

University hospitals are a crucial part in the medical landscape: they should not only provide highly specialized oncological treatment but also be innovative. Being innovative and productive may lead to some tensions, as the implementation or especially development of new treatment approaches or protocols requires time and personnel expansion, which could counteract attempts to increase treatment unit capacities by, e.g., more hypofractionated regimes.

Brachytherapy is one of the subspecialties of radiation oncology in which our study indicated that regional centralization has already begun. This is supported by the increased catchment area of these patients and the relative increase in their proportion in relation to the distance from our department. Brachytherapy plays an undisputed role in the treatment of cervical and endometrial cancer, and is an option for ultra-fractionated, highly conformal definitive treatment in prostate cancer [18]. It has a unique role in radiotherapy, comparable to surgical subdisciplines, as it requires hands-on training and, as with surgery, a correlation between the number of interventions per year and the quality of outcomes is expected. Considering these arguments, brachytherapy might be a good exemplary indication in which centralization may benefit our patients, even if they have to travel longer distances. Furthermore, hands-on brachytherapy curriculums to train residents should be mandatory at these centers [19].

Our study revealed some changes in the distribution of cancer types over the years. As might be expected, the number of patients treated for esophageal cancer decreased, with perioperative FOLFOX being implemented as the preferred treatment option for adenocarcinoma [20]. However, neoadjuvant chemoradiotherapy can still be considered in patients not eligible for FOLFOX. The largest relative and absolute increase in our cohort was observed in cases of skin cancer. These changes correlate with the significant reported increase in the total number of patients diagnosed with skin cancer, particularly non-melanoma skin cancer [21]. To adapt to this trend, we have implemented moulage-based brachytherapy treatment protocols for patients with non-melanoma skin cancer on the face who are not suitable for surgical approaches [22]. As the population ages, it is reasonable to assume that these numbers will increase. Older patients with more comorbidities may be less likely to undergo surgery and more likely to opt for less invasive treatment options, especially those with limited life expectancy [23]. Our brachytherapy protocols deliver acceptable cosmetic and toxicity results and are suitable for anatomical regions that are not ideal for percutaneous radiation delivery [24]. The establishment of this treatment option demonstrates the innovative capabilities of the radiotherapy department and highlights the importance of allocating personnel resources. Nevertheless, metastasis-directed radiotherapy accounted for nearly one out of every five cases. In the past, metastasis-directed radiotherapy was primarily performed with a palliative intention. However, in the area of oligometastatic treatment protocols, the total number of metastasis-directed radiotherapy treatments may increase further, as it is now part of curative treatment approaches and shows clinical meaningful benefits for, e.g., patients with prostate cancer [25].

This study took a pragmatic approach to identifying trends within a large cohort over a long period, based on a raw dataset. However, authorization from the ethics and data protection board substantially delayed the start of the data analysis. Furthermore, the raw data were extracted by the clinical data management organization rather than by the researchers themselves due to regulatory requirements, which resulted in delays when adapting the coding and extent of the raw data provided. Additionally, the aforementioned incorrect ICD association in the raw data led to a time-consuming corrective process. This study therefore illustrates that the authorization process and access to primary data in the context of clinical research, as it is currently regulated, may not be adequate for competing with other research institutions when it comes to big data analysis in the era of artificial intelligence solutions.

### Limitations

Our study has some limitations. Firstly, the study is retrospective in nature. Secondly, patients treated without CT planning scans or not treated with ultrasound-planned HDR brachytherapy for prostate cancer were not eligible for inclusion. However, these patients are very rare in our department, accounting for less than 1% of cases. Furthermore, our cohort does not enable us to capture all trends in SBRT or stereotactic radiosurgery (SRS) since some patients received SBRT or SRS treatments at the CyberKnife Center. Thirdly, our study was conducted in a department that specializes in radiation oncology. Therefore, we cannot provide data on potential changes in the number of benign cases treated on orthovoltage machines, for example. Fourth, we acknowledge that offering new technologies, such as 3D mold HDR brachytherapy, could lead to more referrals to our clinic. However, almost all of these patients were referred by our own dermatology department, and a radiotherapy indication was present, regardless of the available technique. Fifth, the absolute number of patients and the proportion of cancer types could be affected by dynamic demographic changes in the Frankfurt Rhine-Main region. For example, the population of Frankfurt alone increased from 667,925 in 2011 to 743,268 in 2022 [26]. Sixth, the distance to the radiotherapy department is calculated using simplified spherical trigonometry. Travel time for our patients can vary considerably depending on the time of day their treatment is scheduled. Creating a model that considers these differences was beyond the scope of this study.

## Conclusion

In conclusion, our study reveals several trends in radiation oncology. These include an aging patient population and a substantial rise in the number of skin cancer cases. It also shows that, while university radiation departments primarily serve as local radiation providers, they may already offer unique regional expertise in subdisciplines such as brachytherapy. The current regulatory landscape should be critically reviewed to optimize workflow in local, data-driven clinical research.

## Data Availability

Due to current data protection guidelines, the data relating to this article is not publicly available. The data may be requested from the corresponding author upon receipt of a justified request, provided this is approved by the ethics committee or the data protection authority.
